# Interactive Role of the DHPR β_1a_ SH3 Domain in Skeletal Muscle Excitation–Contraction Coupling

**DOI:** 10.3390/biom15111610

**Published:** 2025-11-17

**Authors:** Yamuna Karunasekara, Shouvik Aditya, Nicole C. Norris, Jean Cappello, Angela F. Dulhunty, Philip G. Board, Jose M. Eltit, Claudio F. Perez, Marco G. Casarotto

**Affiliations:** 1 John Curtin School of Medical Research, Australian National University, Canberra, ACT 2601, Australia; 2 The Research School of Biology, Australian National University, Canberra, ACT 2601, Australia; shouvik.aditya@anu.edu.au; 3 Department of Physiology and Biophysics, School of Medicine, Virginia Commonwealth University, Richmond, VA 23298, USA; 4 Department of Anaesthesiology, Brigham and Women’s Hospital, Harvard Medical School, Boston, MA 02115, USA

**Keywords:** dihydropyridine receptor, ryanodine receptor, SH3 domain, excitation contraction coupling, skeletal muscle

## Abstract

Excitation–contraction (EC) coupling in skeletal muscle requires a physical interaction between the voltage-gated calcium channel, dihydropyridine receptor (DHPR), and the ryanodine receptor (RyR1) Ca^2+^ release channel. Although the exact mode of communication that links these two membrane proteins remains to be fully resolved, both the α_1s_ and β_1a_ subunits of DHPR are two of a select number of critical proteins involved in this process. A detailed in vitro interaction study of these two proteins reveals that their association occurs between the β_1a_ SH3 domain and the polyproline motifs located in a critical region of the α_1s_ II-III loop. We demonstrate that subtle changes in the composition of the β_1a_ SH3 domain influences the ability of β proteins to bind to II-III loop proteins and investigate the effect of these changes on EC skeletal coupling. Furthermore, investigation into the composition of the II-III loop shows that previously identified amino acids demonstrated to be important in EC coupling are implicated in in vitro binding. In summary, we ascribe a role for the DHPR β_1a_ which involves the engagement of its SH3 domain with the α_1s_ II-III loop and propose a scenario whereby this interaction may facilitate skeletal muscle EC coupling.

## 1. Introduction

Dihydropyridine receptors (DHPRs) are multi-subunit voltage-gated calcium channels that play an essential role in muscle contraction. In skeletal muscle they are located in the surface membrane and in transverse (t-) tubule invaginations, where they facilitate the conversion of an action potential signal into muscle contraction. This involves a physical interaction transmitted between the DHPR and the ryanodine receptor calcium release channel (RyR1) located in the sarcoplasmic reticulum (SR), enabling the RyR1 channel to release stored Ca^2+^, which subsequently initiates a cascade of events leading to muscle contraction [1]. What is unknown and somewhat controversial is the mechanism by which the transmission of the signal from DHPR to RyR1 occurs.

**Figure 1:**
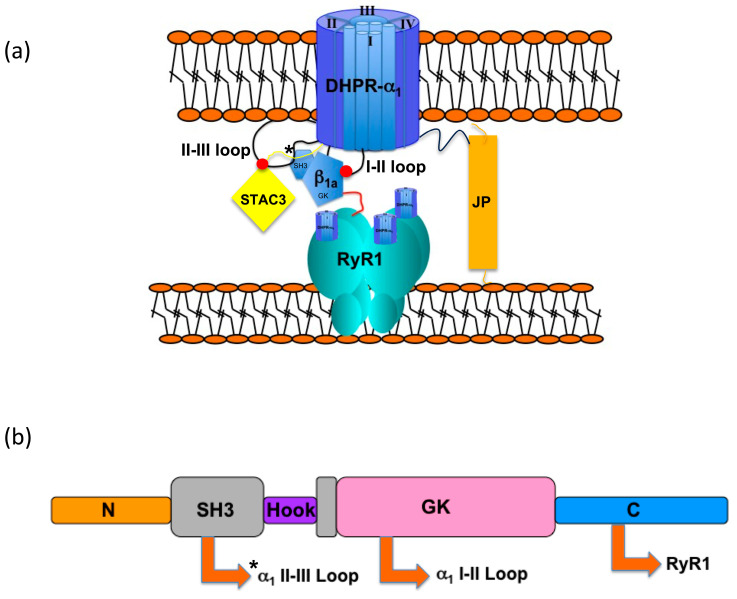
DHPR tetrad/RyR1 tetramer schematic representation depicting key elements involved in skeletal muscle EC coupling: DHPR, β_1a_, RyR1, STAC3, and junctophilin 2 (JP2). (**a**) Diagram expansion outlining the known interactions of DHPR I-II between the α_1s_ and β_1a_ subunit GK domain and between the DHPR II-III loop and STAC3 SH3 domain (depicted as red dots). The asterisk denotes the proposed interaction site between the α_1s_ II-III loop and β_1a_ SH3 domain. The red line depicts an interaction between the β_1a_ C-terminal tail and RyR1(location not yet established). (**b**) The domain architecture of β_1a_ illustrating a split SH3 domain (grey) and a guanylate kinase (GK) domain (pink). The arrows denote known and proposed (*) binding with interaction partners.

It has been established that key features of skeletal muscle EC coupling can be reconstituted in tsA201 cells through the incorporation of five core proteins including RyR1, the α_1_ and β_1a_ subunits of DHPR, STAC3, and junctophilin (JP) [2] (see Figure 1). RyR1, the α_1_ and β_1a_ DHPR subunits, and STAC3 are well-known for their involvement in skeletal muscle voltage-induced Ca^2+^ release [3,4,5], while JP1&2 promotes the formation of endoplasmic reticulum–plasma membrane junctions [6,7].

DHPR alpha (α_1s_) is the largest of the five subunits that makes up the DHPR complex and acts as the voltage sensor. It consists of four transmembrane repeats that are connected by cytoplasmic loops, one of which, the II-III loop, is crucial for skeletal muscle EC coupling to take place [8]. Within this loop lies a critical region (C region, residues 720–765) in which key residues have been identified as key determinants driving skeletal muscle EC coupling [8,9,10] (Figure 1a). A widely held view is that the C region of the II-III loop must somehow communicate or promote communication with the RyR1 for it to open and conduct Ca^2+^ [1]; however, conjecture remains as to whether direct binding must take place or an alternative pathway is involved. This question was partially addressed in a crystallographic and binding study that showed an in vitro interaction between the C region of the II-III loop and the SH3 domains of STAC3 [11]. However, the specific region of the II-III loop C region reported to interact with STAC3 (residues 747–760) [11] did not correspond to an upstream region (residues 739–744) within the II-III loop C region previously determined to be important for EC coupling [12]. This begs the question as to whether the II-III loop C region may engage with multiple binding partners.

In addition to the α_1s_ subunit, the DHPR beta subunit, β_1a_, has also been shown to be a central player in skeletal muscle EC coupling. As with other β-subunit isoforms, β_1a_ plays a dual role as a chaperone by targeting the α_1_-subunit to the membrane and also as a modulator of the DHPR α_1_-subunit [13,14]. The cytoplasmic β-subunit is made up of a core Src-homology 3 (SH3)-guanylate kinase (GK) module, with the SH3 domain split by a HOOK (Figure 1b) [15,16,17]. The core SH3 and GK domains are highly conserved among all four genes that encode β-subunits, with the N- and C-termini and the HOOK region showing the greatest sequence diversity [18]. In an attempt to map the roles of the individual modules, domain swapping experiments involving different isoforms have been reported. In mouse myotubes and zebra fish, the C-terminal domain of β_1a_ was identified as a common determinant for EC coupling in skeletal muscle [19,20,21], while in zebra fish, the SH3 domain was noted as important in voltage sensing [22]. As there are potential SH3-binding motifs in the critical II-III loop region, and because of the involvement of the α_1s_ II-III loop and β_1a_ in EC coupling, we have investigated the possible interaction of the β_1a_ subunit with the α_1s_ II-III cytoplasmic loop and its polyproline rich elements.

The core structures of all four different β-subunit isoforms (β_1a_, β_2a_, β_3_, and β_4_) have been solved by X-ray crystallography, and all isoforms have very similar structures that resemble a family of membrane-associated guanylate kinase (MAGUK) proteins [15,16,17,23]. The α_1_ and β DHPR subunits bind through a conserved, high-affinity interaction (~3–50 nM) between the alpha interaction domain (AID) in the I-II loop of the α_1_-subunit and an AID-binding pocket (ABP) in the GK domain of the β-subunit [17,23,24,25]. In addition to this high-affinity interaction, other lower affinity β-subunit interaction sites are also purported to exist [18]. What remains to be determined is whether there are other interaction partners and the role that they may play in skeletal muscle EC coupling. The binding sites of canonical SH3 have been extensively studied [26]; however, the unique structural architecture of MAGUK SH3 domains [18] raises the possibility of a non-canonical mode of binding.

We have utilized fluorescence quenching experiments to characterize the interaction between the α_1s_ DHPR II-III loop and β_1a_ subunit elements in vitro. The strategy involved the identification of interacting segments/domains within these proteins and probing their role through substitution with comparable sequences derived from cardiac isoforms, i.e., α_1c_ and β_2a_. We explored a series of α_1s/_ α_1c_ DHPR II–III loop peptides and selected β_1a_/β_2a_ chimeras with the aim of identifying and consolidating the specific regions that confer skeletal isoform-specific characteristics. Myotube electrophysiology techniques have been employed to examine skeletal muscle EC coupling using selected chimeras. Taken together with other previously identified interactions, our data provides valuable, unexplored insights into the roles the DHPR β_1a_ subunit and II-III loop play in skeletal muscle EC coupling.

## 2. Materials and Methods

The mouse β_1a_ and β_2a_ subunits (NCBI reference sequences—NM_031173 and NM_023116), the β_1a_-SH3 domain (β_1a_ residues V101-P272), and the α_1s_ II-III loop (NCBI reference sequence—X05921, residues E666-L791) were amplified by PCR. Each PCR product was cloned in-frame downstream of a polyhistidine-tagged ubiquitin sequence in the plasmid pHUE [27]. The plasmids were transferred into *Escherichia coli* BL21, and expression of fusion protein was induced by addition of 0.1 mM IPTG (isopropyl β-D-1thiogalactoside) to the culture medium. The His-tagged proteins were purified by chromatography on Ni-agarose. Ubiquitin was removed from the N-terminal end of the recombinant proteins by digestion with a His-tagged ubiquitin-dependant protease [27]. The recombinant β-subunit proteins and the II-III loop were further purified by preparative electrophoresis using a Bio-Rad model 491 prep cell. The final samples were dialysed into a buffer containing 50 mM sodium phosphate pH 8 and 300 mM NaCl (buffer A) and stored at −70 °C.

The β_1a_ core construct (β_1a-core_) (residues 68–185 and 252–462) was purchased from DNA 2.0, Newark, USA. The gene was inserted into pJ411^kanR^ containing a pUC origin of replication, a T7 promoter, and an N-terminal hexahistidine tag containing an HRV 3C protease site. The protein was expressed in BL21 (DE3). Bacteria were cultured in 2 × YT supplemented with yeast nitrogen base without amino acids (1.34 g∙L^−1^) and kanamycin (50 µg∙mL^−1^). Cells were cultured to an optical density (OD_600 nm_) of~0.3 at 37 °C and the temperature reduced to 16 °C. Protein expression was induced with 0.2 mM IPTG at an OD_600 nm_~0.6 for 16 h. The cell pellet was resuspended in 50 mM sodium phosphate at pH 7.0, 500 mM sodium chloride, and 30 mM imidazole. Cells were chemically lysed with the addition of lysozyme (1 µg∙mL^−1^), DNaseI (1 µg∙mL^−1^), and RNaseH (1 µg∙mL^−1^). Cells were physically lysed with the French Pressure Cell. Cleared lysate was applied to HisTrap (GE Healthcare) column equilibrated in buffer A and washed with buffer A. Then the column was washed with buffer A containing 1.5 M sodium chloride before being returned to buffer A. Finally, the protein was eluted with a gradient of imidazole. The eluate was simultaneously dialysed into buffer A, and the tag was cleaved overnight with HRV 3C protease (Pierce Scientific, Rockford, IL, USA) at 4 °C. The eluate was reapplied to the HisTrap column and the flowthrough dialysed into 20 mM Tris, 200 NaCl at pH 7.0. The protein was concentrated to ~8 mg/mL.

The β_1a_ SH3 protein (residues V101-P272) was purified by IMAC (immobilized metal affinity chromatography) under denaturing conditions (6M guanidinium HCl) using Ni-NTA resin. The purity of the protein was assessed by SDS page gels (Supplementary Figure S1). The purified protein was then refolded by dialysing against buffer A, and the folded structure was checked by circular dichroism (Supplementary Figure S2). The purified and refolded protein was stored (−70 °C) at concentrations of ~1μM. It was not possible to directly assess the aggregation state of the β_1a_ SH3 protein construct at 1μM as the SEC UV peak absorbance was below detectable levels. However, the fact that the fluorescence binding results for the full-length β_1a_ and SH3 domain construct are virtually the same for all experiments (see Table 1) suggests that the mode of binding is similar and the SH3 domain construct is most likely in a monomeric state.

### 2.1. Peptide Synthesis

Peptides were synthesized by the Biomolecular Resource Facility of the John Curtin School of Medical Research (Australian National University, Canberra, Australia) using an Applied Biosystems 430A peptide synthesizer and purified by reverse phase high-performance liquid chromatography (RP-HPLC) on a Jupiter 300 C4 column. Peptides were eluted using a linear gradient from buffer A (deionised water and 0.1% trifluroacetic acid, TFA) and buffer B (acetonitrile and 0.1% TFA). Purified peptide fractions were identified by mass spectroscopy using an AB MDS Sciex 4800 MALDI-TOF-TOF Mass Analyzer. The peptide sequences were derived from NCBI reference sequence NP_001095190.1 (skeletal) and NP_001129994.1 (cardiac).

### 2.2. Fluorescence Quenching Experiments

Equilibrium dissociation constants for β_1a_ and β_2a_ subunit and β_1a_-SH3 domain binding to the α_1s_ II-III loop and its peptides were determined with a Perkin-Elmer LS 50B6 Spectrofluorimeter. Changes in the intrinsic fluorescence of the tryptophan residues of the β_1a_ subunit or the β_1a_-SH3 domain was monitored while titrating the α_1s_ II-III loop or its peptides. Excitation was at 280 nm, and fluorescence emission was monitored at 340 nm. Data was corrected for dilution effects and for intrinsic fluorescence of the II-III loop or its peptides and fitted with nonlinear regression using GraphPad Prism software (6.0), as previously described [28].

### 2.3. Circular Dichroism (CD)

CD data was aquired on a Chirascan™ Circular Dichroism Spectrometer (Applied Photophysics Ltd., Leatherhead, UK). Samples were prepared at a concentration of <10 μM in 10 mM NaF buffer, pH 8.0. Spectra were measured at 20 °C over the range of 180–320 nm at a scan rate of 1 nm/s. For all measurements, a cell with 0.1 path length was used. CD spectra were corrected for buffer contributions, and an average of three scans was subjected to a smoothing function using the proprietary software of the manufacturer.

### 2.4. cDNA Constructs and Virus Packaging for Myotube Studies

Cloning and expression of the full-length cDNA of mouse DHPR β1a subunit (GenBank NM_031173) into the bicistronic retroviral vector pC-MMP-MCS-IRES-Puro were described previously [29]. Insertion of the mouse β2a RT loop (GeneBank, NM_199247.2) was introduced into the homologous loop of the β1a mouse gene using Gibson assembly (New England Biolabs) and double-stranded DNA fragments (gBlocks, Integrated DNA Technologies). The presence of substitution and proper reading frame of all selected clones were confirmed by sequencing before use. Virus packaging was performed with a set of three packaging vectors, as described elsewhere [29].

### 2.5. Isolation of Primary Myotubes, Cell Culture, and Calcium Imaging

All animal procedures were carried out in accordance with the guidelines of Harvard Medical Area Standing Committee on Animals under experimental protocol number 2589, approved on 01 Sept 2014. Primary myoblasts from DHPRβ1a-null mouse skeletal muscles were isolated from the hindlimb of neonatal mice, as described elsewhere [30,31]. Stably transduced cell lines were then obtained by infection of primary β-null myoblasts with virion particles carrying the β1a genes at a multiplicity of infection (MOI) of 0.5 and then selected with 1.6 g/mL Puromycin for 2 weeks, as described previously [32]. Cells expressing mouse Wtβ1a or β1a-loop chimera subunit were then grown and differentiated in 96-well plates, as described previously [33]. Calcium imaging was performed 5–6 days after differentiation in myotubes loaded with 5μM Fura-2-AM or 2μM Fluo-4 (Molecular Probes) in imaging buffer (125 mM NaCl, 5 mM KCl, 2 mM CaCl2, 1.2 mM MgSO4, 6 mM glucose, and 25 mM HEPES-Tris, pH 7.4). Cell membrane depolarization was performed by 5s of perfusion with 5–7 volumes of imaging buffer containing increasing concentrations of KCl. To preserve osmolarity of the depolarization buffer, the increased K concentration was compensated with an equivalent reduction in total NaCl concentration. Sarcoplasmic reticulum Ca^2+^ content of Fura-2-AM-loaded cultured myotubes was estimated from the peak fluorescent amplitude of the Ca^2+^ transient induced by 40 mM caffeine stimulation in the presence of 1μM thapsigargin (Tg) and in the absence of extracellular Ca^2+^ [34,35]. Extracellular Ca^2+^ entry at rest was estimated from the rate of dye quench by Mn^2+^ entry in myotubes loaded with 5μM Fura-2-AM in the presence and in the absence of 10μM KB-R7943 (glycerol, 2-(2-(4-(4-nitrobenzyloxy) phenyl)ethyl)-isothiourea methane sulfonate), as described previously [35,36]. Cells were then imaged at 360 nm (5 nm) with an intensified 10-bit digital CCD camera (XR-Mega-10, Stanford Photonics, Stanford, CA, USA) using aDG4 multiwavelength light source. Fura-2 images were acquired with a 40 objective (Olympus, UApo/340), and fluorescent emission at 510 nm was then captured from regions of interest within each myotube at 33 frames/s using Piper-controlled acquisition software (Stanford Photonics) and is expressed as the ratio of signal collected at alternating 340 nm/380 nm excitation wavelengths. For Mn^2+^ quench studies, images were acquired at 510 nm as the average of 5 individual recordings captured at 6 frames/s. Fluo-4 fluorescent images were acquired using a 60 objective, and emission at 510 nm was then collected from regions of interest from individual cells excited at 490 nm and is expressed as arbitrary fluorescent units.

### 2.6. L-Type Ca^2+^ Current Determinations in Cultured Myotubes

The macroscopic Ca^2+^ current was determined using the same equipment and protocols previously described (PMID: 29212769). The Sylgard-coated patch pipettes had a resistance of ~2 MΩ when filled with the pipette solution. The internal solution consisted of 140 mM Cs-Aspartate, 5 mM MgCl_2_, 10 mM Cs-EGTA, and 10 mM HEPES titrated with CsOH to pH 7.2. The composition of the external bath solution was 145 mM TEA-Cl, 10 mM CaCl_2_, and 10 mM HEPES titrated with TEA(OH) to pH 7.4. Whole-cell parameters (mean ± SE) were as follows: Cm (pF) = 68.7 ± 5.26 and 74.8 ± 5.94; Rm (GΩ) 2.67 ± 0.320 and 2.09 ± 0.217; and Ra (MΩ) = 5.6 ± 0.28 and 5.8 ± 0.27 for Wt-β1a (N = 22) and β1a-RT-loop (N = 21) myotubes, respectively.

The steady-state voltage dependence of the Ca^2+^ currents was fitted to the following equation:

I (Ca) = Gmax (V − Vr)/{1 + exp[(V_1/2_ − V)/k]}, where Gmax is maximal conductance, V corresponds to the test potential, V_1/2_ is the potential at which G = 1/2 Gmax, k represents a slope parameter, and Vr is the reversal potential. These parameters are included in Table 2.

## 3. Results

The DHPR II-III loop (residues 666–790, NCBI reference sequence NP_001095190), full-length β_1a_ (β_1a-FL_)_,_ β_2a_ (NCBI reference sequences—NM_031173 and NM_023116), β_1a_ and β_2a_ mutants and chimeras, β_1a_ SH3 domain (β_1a-SH3_, residues 101–272), and the β_1a_ core (β_1a-core,_ residues 68–185 and 252–462) were all expressed and purified to homogeneity (Supplementary Figures S1 and S2).

**Figure 2:**
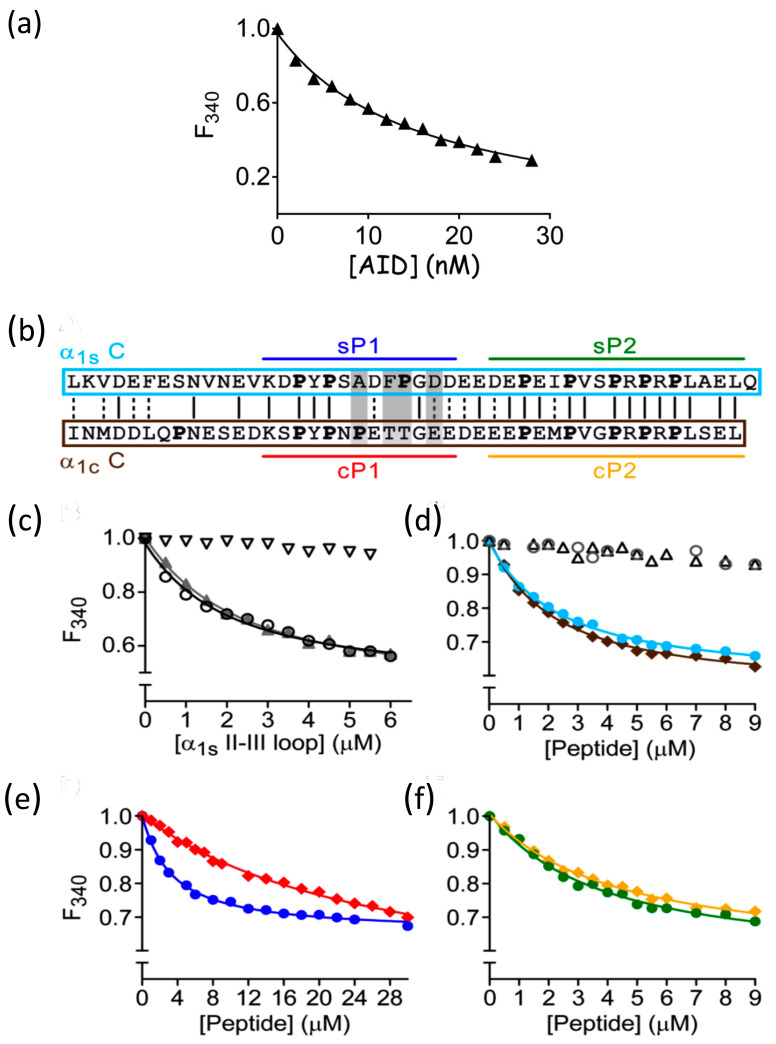
Fluorescence binding measurements for DHPR β_1a_. (**a**) Fluorescence binding curve for β_1a-FL_ and DHPR α_1s_ I-II loop (AID) (▲). The relative fluorescence of β_1a-FL_ at 340 nm (F_340_) is plotted as a function of AID peptide concentration. The concentration of β_1a-FL_ in the cuvette was 50 nM. (**b**) Sequence alignment between the skeletal (α_1s_) and cardiac (α_1c_) II-III loop C region. Two polyproline SH3 binding regions are highlighted, sP1/sP2 and cP1/cP2, corresponding to DHPR α_1s_ and α_1c_, respectively. The solid lines indicate conserved residues and dotted lines amino acid types with similar properties. The grey shading denotes residues that have been previously mutated and identified as important with respect to EC coupling [12]. Relative fluorescence of β_1a-FL_ and/or its β_1a-SH3_ at 340 nm (F_340_) are plotted as a function of II-III loop peptide concentration. (**c**) Binding curves between β_1a-FL_ (▲) and β_1a-SH3_ (○) and the DHPR II-III loop. BSA was used as the control experiment (∇). (**d**) Binding curves of the α_1s_ C (●)(residues 720–765) and α_1c_ C (◆)(residues 851–895) II-III loop C-region peptides with the β_1a-FL_. Control binding experiments included the scrambled skeletal C region (∆) and proline to alanine mutant (○) peptides, respectively. (**e**) Binding curves of the sP1(●) and cP1 (◆) peptides with the β_1a-FL_. (**f**) Binding curves of the sP2 (●) and cP2(◆) peptides with β_1a-FL_. The coloured bars above in panel 2a denote the location of peptide segment within the II-III loop C region.

The most characterized interaction between DHPR β subunits and the DHPR α_1_ I-II loop to date involves the AID region binding to the DHPR β subunit with nanomolar affinity [24,25,37]. Because full-length DHPR β subunits are prone to aggregation [37], the majority of these previous studies have utilized truncated or modified β subunit constructs. In the case of the full-length DHPR β subunit, this makes some of the more common binding techniques such as Isothermal Titration Calorimetry (ITC) impracticable. However, because of the intrinsic fluorescence properties of full-length β_1a_ and β_2a_, their interactions with α_1s_-subunit fragments can be measured using sub-micromolar concentrations of the β subunits, thereby minimizing protein aggregation. Fluorescence binding (or fluorescence quenching) is a highly sensitive technique that has been used extensively in our studies and by others to probe protein–protein interactions [28,38,39]. The affinity of AID peptide and full-length β_1a_ was measured, with fluorescence emission monitored at 340 nm. After correction of the data for dilution effects and intrinsic fluorescence of the AID peptide, a titration curve was generated (Figure 2a) and fitted using an exponential-based equation [28] to yield a dissociation constant (K_d_) of 15.2 ± 1.8 nM, which falls within the previous range of results using truncated versions of the DHPR β subunit [24,25] and indicates that full-length β_1a_ is correctly folded.

Therefore, a series of fluorescence binding experiments were performed with the β_1a_ to examine its potential binding affinity with the DHPR α_1s_ II-III loop and its peptide segments. The results are displayed in Figure 2 and summarized in Table 1. Significantly, a K_d_ value of 2.5 ± 0.4 μM was observed between β_1a_ and the DHPR II-III loop. A comparable affinity (2.6 ± 0.2 μM) was observed for the II-III loop binding to the β_1a_ SH3 domain (β_1a-SH3_, Figure 2c and Table 1), indicating that the II-III loop binds β_1a_ primarily through its SH3 domain. The observed micromolar binding constants are typical for polyproline–SH3-mediated interactions, which have dissociation constants in the micromolar to millimolar range [26].

To pinpoint the exact regions of the II-III loop that bind to β_1a_, the proline-rich C region (720–765) of the loop (α_1s_ C) was synthesized and its ability to bind to β_1a_ and β_1a-SH3_ measured (Figure 2d). Because the II-III loop is an intrinsically disordered protein [28], the structural integrity of the C region was likely to be minimally disrupted by its excision from the loop. Binding affinities of 4.9 ± 0.6 and 3.6 ± 0.6 μM were found for β_1a_ and β_1a-SH3_, respectively (Table 1), suggesting that α_1s_ C is responsible for binding to the SH3 domain of β_1a_. To exclude non-specific binding events, extensive control experiments were performed with a scrambled C-region peptide and a mutant C peptide, where all of the proline residues were substituted for alanine residues (Figure 2d). In both cases, no measurable binding was detected, highlighting the overall importance of the presence and the location of the proline residues.

The amino acids that make up the critical region of the skeletal II-III loop contains a PxxDY motif (residues 733–745) as well as two adjacent PXXP motifs (residues 748–764, Figure 2b), all of which are potential candidates for SH3 domain recognition [26,40]. Therefore, two shorter peptides spanning these regions were synthesized and denoted as peptides sP1 and sP2, respectively (Figure 2b), and tested against β_1a_ and β_1a-SH3_. Both sP1 and sP2 peptides bind to β_1a_ and β_1a-SH3_ in the low micromolar range (3–5 μM, Table 1), demonstrating that one or both of these regions contribute to the C-region–β_1a_ SH3 interaction.

In previous dysgenic (α_1s_-null) mouse myotube studies, the sP1 region was identified as being of critical importance in EC coupling [8,10]. Substituting residues A739, F741, P742, and D744 with their cardiac equivalent amino acids markedly altered the skeletal voltage-dependent Ca^2+^ release profiles [12]. These residues are contained in the sP1 peptide, prompting us to explore the binding of the cardiac homologue of the sP1 peptide (cP1) to the β_1a_ SH3 domain. The affinity of the cP1 peptide for β_1a_ and β_1a-SH3_ was reduced compared to sP1 (4 to 7-fold, Figure 2e, and Table 1). We also found that, of the four variant residues, P742 and D744 in sP1 were independently responsible for the reduction in binding (Table 1). In contrast, the cardiac homologue of sP2 and cP2 displayed a similar affinity to its skeletal counterpart (Figure 2f). Notably, in myotube experiments, the substitution of cP2 on a skeletal II-III loop background did not impact EC coupling [12]. This result is in line with the high sequence similarity of the skeletal and cardiac P2 region compared to that observed in the P1 region (Figure 2b) and is therefore suggesting that the SP1 region of the skeletal II-III loop is most likely responsible for the molecular recognition of the β_1a_ subunit.

**Figure 3:**
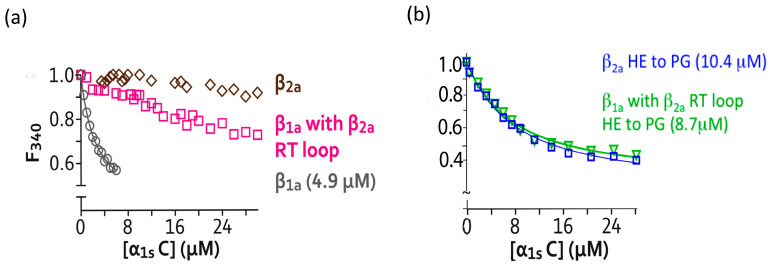
Fluorescence binding measurements for DHPR β_2a_, β_1a_, and β_2a_/β_1a_ chimeras to α_1s_ C peptide. (**a**) Binding curves for β_2a_ (◇) and the β_1a_/β_2a_ RT-loop chimera (□) with peptide α_1s_ C. Binding between β_1a-FL_ and α_1s_ C is shown for comparison (○). (**b**) Curves for β_2a_ (□) and the β_1a_/β_2a_ RT-loop chimera (∇), where β_2a_ RT-loop residues (H&E) have been mutated to β_1a_ residues (P&G), binding to peptide α_1s_ C.

In light of the interaction profiles associated with the β_1a_ subunit, it was of interest to examine whether these α_1s_ polyproline-rich peptides could bind to the cardiac β_2a_ isoform. Figure 3a shows that no measurable binding was detected between β_2a_ and α_1s_ C. A comparison of the SH3 domain sequences for each of the four β isoforms reveals that, despite good overall sequence conservation, there is significant variation in the β_1a_ RT loop relative to the other isoforms [37]. Therefore, a β_1a_ chimera was designed with the β_1a_ RT loop replaced with the β_2a_ RT loop. Accurate fitting of a binding curve was not possible; however, it is clear there is weak, residual binding of this chimeric protein to α_1s_ C (Figure 3a).

**Figure 4:**
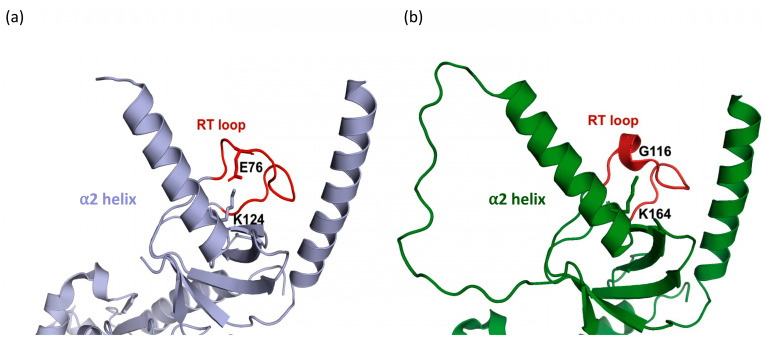
Interactions of β_2a_ and β_1a_ SH3 RT loops. (**a**) The X-ray crystal structure of the SH3 domain of rabbit β_2a_ (mauve, PDB code1t3l). The α_2_ helix and the RT loop interact through a salt bridge involving the side chains of E76 and K124. (**b**) The X-ray crystal structure of the SH3 domain of rabbit β_1a_ (green, PDB code 4zw2), showing the absence of a salt bridge due to the existence of an RT-loop glycine residue (G116).

In three of the β-subunit structures, β_2a_, β_3_, and β_4_, the α2 helix and RT loop are linked via a salt bridge [15,16,17] (Figure 4a). The presence of this salt bridge stabilizes these two structural elements so as to position them over and occlude the α_1s_ C binding site region. The participating positively charged residue in the α2 helix is conserved in all of these three β subunit isoforms. However, in β_1a_, the equivalent negatively charged residue in the RT loop is replaced by a glycine and cannot form an equivalent salt bridge (Figure 4b). Therefore, the residues P115 and G116 present in the RT loop of β_1a_ were reintroduced into the β_2a_ protein as well as into the weakly binding chimeric β_1a_ protein (β_1a_ background with β_2a_ RT loop). In both instances, binding of the α_1s_ C peptide was enhanced with affinities of ~ 8 and 10 μM (Figure 3b, Table 3).

### Calcium Release and L-Type Channel Function

**Figure 5:**
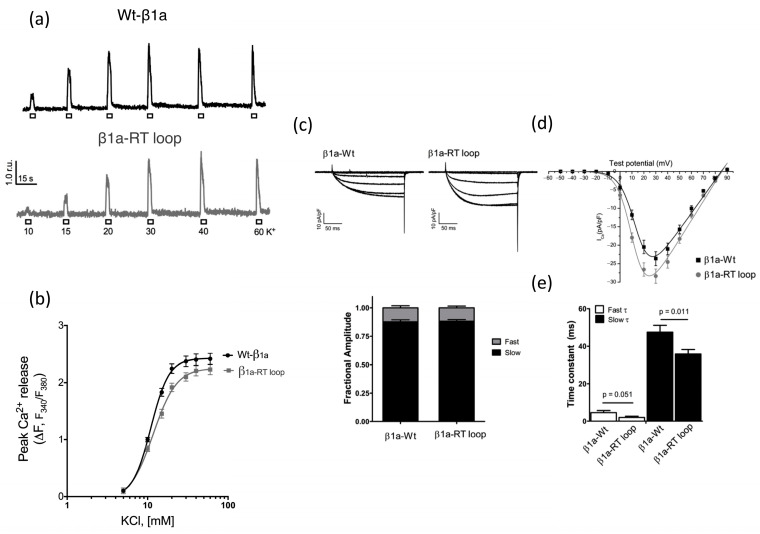
Ca^2+^ release and L-type Ca^2+^ current patch clamping measurements. (**a**) Depolarization-induced Ca^2+^ release of β_1a_ subunit Wt and RT-loop constructs. Representative fluorescent traces of K-dose responses from Wt- and RT-loop-expressing myotubes. (**b**) Average peak fluorescent amplitude of KCl-induced Ca^2+^ release transients observed in Fura-2 loaded myotubes expressing Wt (black) and RT loop (grey). Data are expressed as mean SE and were fitted to a sigmoidal dose–response curve. (**c**) Representative traces of the L-type Ca^2+^ current in β-null myocytes expressing the β_1a_-Wt or β_1a_-RT-loop constructs. (**d**) Comparison of current –voltage relationship (I-V), myocytes where depolarized from a holding potential of −50 mV, and 200 ms pulses were applied from −40 mV to +90 mV in 10 mV increments. (**e**) Current kinetics were determined by fitting the current recorded at +30 mV (maximal current density) to a biexponential equation; fractional amplitude of the fast and slow components of the current were identical between cell types, but the onset of the current was faster for the β_1a_-RT-loop-expressing myotubes. Data is presented as mean ± SE of n = 22 (Wt-β1a) and n = 21 (β1a-RT loop) recordings of at least 3 independent days.

The effect of swapping the β_1a_ RT loop for its equivalent counterpart, β_2a_, was analyzed using depolarization-induced Ca^2+^ release and whole-cell patch clamp experiments in cultured myotubes. Figure 5a,b, show representative KCl dose–response curves of Fura-2-AM-loaded for myotubes expressing either WT- or the β_1a_-RT-loop construct. Investigations of these phenotypes indicate that there is a modest but discernible reduction in maximal peak Ca^2+^ amplitude and a shift in the EC50 value from 6.68 ± 0.45 mM (WT) to 9.42 ± 0.53 mM for the RT loop. The level of SR calcium load of cultured cells was also compared and showed that the RT-loop mutation did not affect the SR calcium content.

Whole-cell patch-clamp techniques were employed to examine the effects of the β_1a_ RT-loop mutation on DHPR channel function. Comparison of Ca^2+^ current recording shows that WT-β_1a_ and β_1a_-RT-loop-expressing myotubes display characteristically slow-activating Ca^2+^ currents (Figure 5c). Further analysis revealed β_1a_-RT-loop-expressing myotubes displayed Ca^2+^ currents that were higher than WT-β_1a_ myotubes in peak amplitude magnitude of L-type conductance (Gmax) (Figure 5c,d). In addition, average voltage dependence of the channel activation (V_1/2_) in β_1a_ RT-loop-expressing myotubes was shifted by ~4 mV to more negative potential (Figure 5d and Table 2, *p* = 0.0051). Further analysis of the current kinetics of WT-β_1a_ and β_1a_-RT-loop-expressing myotubes were studied by fitting currents to a biexponential curve and the corresponding slow and fast values at 30 mV plotted as means SE (Figure 5e). A key point of difference between the two myotubes is that the channels activated more quickly in the β_1a_-RT-loop-expressing myotubes.

## 4. Discussion

In contrast to other DHPR β-subunit isoforms, β_1a_ is expressed only in skeletal muscle, where it is an exclusive partner of DHPR α_1s_. It is essential in the functional assembly of skeletal muscle triads and is required to form DHPR tetrads [41]. It has been long recognized that the I-II loop of the DHPR α-subunit is capable of a high-affinity interaction with all beta isoforms [24,25], but it has been flagged that other secondary binding sites may also exist for the SH3 and GK subunits [42]. While the β_1a_ sequence of the N and C-termini and the hook regions are quite distinct from other β isoforms, the overall SH3 domain sequence is conserved; however, within this domain, the RT-loop region shows the greatest sequence variability [37]. This led us to speculate that the properties of this loop may confer different binding properties for the various beta isoforms. In this study, we probed the in vitro binding of the polyproline-rich DHPR II-III loop of the α_1s_ subunit with β_1a_.

By performing in vitro fluorescence binding experiments, we have measured affinities between the β_1a_ SH3 domain and two polyproline regions located on the α_1s_ II-III loop. One of these regions (sP1) has been previously identified in mouse myotube studies as a central component associated with EC coupling [12]. We showed that the equivalent cardiac segment (cP1) binds with lower affinity to β_1a-FL_ compared to its skeletal counterpart (sP1) and that the equivalent cardiac single point mutations, P742 and D744, in sP1 resulted in reduced binding affinities, consistent with the myotube data showing that these residues affected orthograde coupling [12]. It is noteworthy that human mutations involving the residue P742 (P741Q/S) were reported to cause congenital myopathy [43], indicating a physiological impact for this residue.

The second region of interaction involves two contiguous PXXP motifs that are present in both skeletal and cardiac isoforms of the II-III loop (sP2 and cP2). In myotube studies, interchanging sP2 and cP2 did not impact orthograde coupling [12]. The fact that the α_1c_ region of the II-III loop cannot substitute for α_1s_ in reconstituting skeletal muscle EC coupling [9] may indicate that both regions (sP1 and sP2) are required for skeletal muscle EC coupling. Indeed, the sP2 region has been shown to be the primary binding region for the SH3 domains of STAC3 [11], suggesting that the critical C region of the II-III loop may have multiple interaction sites. Interestingly, the fact that, under in vitro conditions, the binding of sP2 and sP1 to β_1a_ both occur highlights the promiscuous nature of polyproline elements binding to SH3 domains—a feature not uncommon for the SH3 binding domain [44]. The precise sequence of binding events leading up to and during EC coupling remains to be determined; however, one can speculate that, in an in vivo setting, there is potential for the polyproline motifs in the II-III loop C region to interact with multiple partners.

Another question that is addressed in this study is whether the composition of the β_1a_ SH3 domain plays an isoform-specific role in the in vitro interaction of the II-III loop. One notable difference between the β_1a_ and RT loops of other isoforms is the presence of Gly116 (β_1a_—numbering), which is substituted in the other isoforms by a conserved Glu or Asp residue. In some of the X-ray crystal structures complexed to the AID peptide, these acidic acid residues participate in the formation of a salt bridge to a conserved basic residue located in the α2-helix and most likely contributes to the stabilization of the occluded polyproline binding site [37]. The absence of this salt bridge in the β_1a_ isoform is likely to increase the dynamic mobility of the RT loop and the α2 helix and enhance its ability to bind to the polyproline motifs. Indeed, binding to α_1s_ C was increased to 8–10 μM when the His-Glu residues in β_2a_ and the β_1a_/β_2a_ RT-loop chimera were replaced by the corresponding Pro-Gly residues in β_1a_. This enhancement in binding is not unprecedented, with a previous report showing that the introduction of a glycine residue within an SH3 domain RT loop enhances its flexibility and increases the domain’s affinity to its polyproline binding partner [45].

There are multiple factors that may influence the accessibility of the β_1a_ SH3 binding site. For instance, there are some very obvious differences between the constructs used for crystallization and other studies. The beta constructs used for crystallization have been heavily modified to promote crystal formation relative to full length β_1a_. Notably, two of the truncated regions in the crystal structure constructs (N-terminal and hook) are directly adjacent to the α1 and α2 helices, which may impact their conformation and SH3 domain binding. Indeed, varying the composition of the hook region of a MAGUK protein has been shown to alter the binding properties of the GK domain via the SH3 domain [46]. It was concluded from this that the SH3 modulates the GK domain allosterically to control its function.

Despite numerous attempts to crystallize a truncated version of β_1a_ by itself, crystallization was only possible in the presence of a CaV AID peptide [37], whereas AID was absent for full-length β_1a_ fluorescence binding experiments. The potential for allosteric changes elicited by AID binding has been flagged in our previous study, demonstrated by a major increase in the melting temperature of β_1a_ arising from the addition of AID [37]. This increase in melting temperature was affected by the substitution of the β_1a_ SH3 RT loop with that of β_2a_, suggesting that conformational changes at one end of β_1a_ can be transmitted throughout the molecule. Moreover, the binding affinity of the AID peptide region was shown to increase by a factor of 2 to 3-fold [37] when the β_2a_ SH3 domain was substituted with that of β_1a_. What can be concluded from this series of observations is that, despite the fact that AID binding occurs exclusively through contact with the GK domain of β_1a_, modification or binding with the SH3 domain can allosterically influence the nature of this interaction.

**Figure 6:**
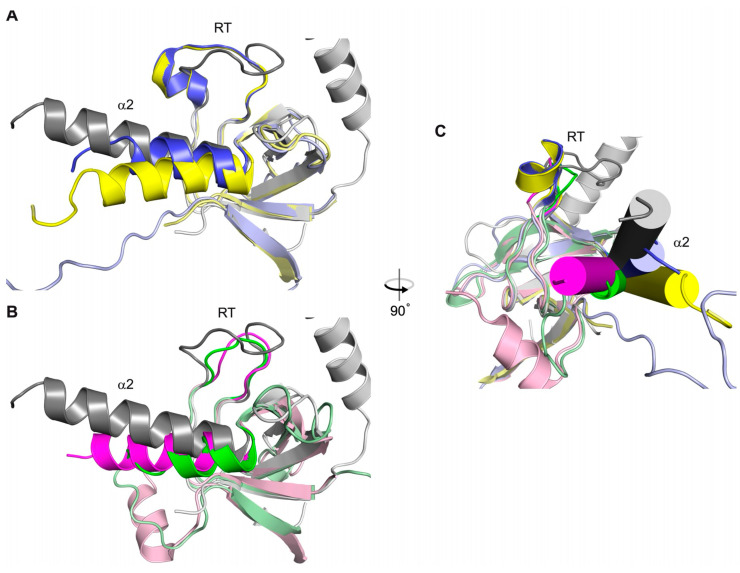
Structural comparison of MAGUK SH3 domains. The SH3 domain of β_2a_ (grey, pdb code 1t3l) was aligned with the SH3 domains of either (**A**) two PSD-95 structures (yellow and blue, pdb code 1jxo and 1kjw, respectively) or (**B**) ZO-1(green, pdb code3lh5) and ZO-3 (magenta, pbd code 3kfv). In (**A**,**B**) for all structures, the α2 helix and RT loop are dark coloured, the rest of the SH3 domain is light. (**C**) SH3 β_2a_ domain aligned with those of PSD-95, ZO-1, and ZO-3, with the structures rotated 90° with respect to (**A**,**B**). The helix is shown as a cylinder. Colouring is the same as for (**A**,**B**).

Comparison of DHPR β_1a_ with other members of the MAGUK family, PSD-95, and ZO-1 reveals that their SH3 binding sites are capable of binding PXXP motif-containing proteins [47,48,49]. In the crystal structures of PSD-95 and ZO-1 so far determined [50,51,52], non-covalent interactions (observed in the β-isoforms) connecting the RT loop and the α2 helix are absent, and, importantly, the RT loop does not occlude either SH3 binding site. Also, the position α2 helix of PSD-95 and ZO-1 is highly variable compared to the beta structures, indicating that this helix is mobile (Figure 6). Therefore, the SH3 domains of PSD-95 and ZO-1, which are functionally and structurally similar to β_1a_ SH3, also support the alternative positioning of both the RT loop and α2 helix, indicating that the polyproline motif binding site can become exposed for this family of proteins.

### 4.1. Functional Roles of the β_1a_ SH3 Domain in Skeletal Muscle EC Coupling

The in vivo roles of the β_1a_ SH3 domain have been previously explored by swapping this domain with that of other isomers. One of the early DHPR beta myotube studies [21] reported that simultaneously interchanging the N-terminal, hook region, and SH3 domain of β_1a_ with those of β_2a_ resulted in no discernible difference in skeletal-like EC coupling. In another study carried out in zebra fish [22], SH3 domain substitution (β_1a_ to β_3_) resulted in a substantial reduction in charge movement and calcium release (>50%), while a comparable study involving substitution of the β_4_ SH3 domain resulted in no detectable effect on charge movement or calcium release [20]. In this study, we have shown in myotubes that on a β_1a_ background, substitution of the β_1a_ SH3 RT loop with that of β_2a_ resulted in a subtle but detectable change in the calcium release profile as well as an effect upon the DHPR channel function. For these two sets of experiments, these changes included a decrease in maximal Ca^2+^ release, while an increase was observed in G_max_ for the Ca^2+^ current experiments. From these results, the data suggest that swapping the β_1a_ SH3 RT-loop segment has only minor effects for both orthograde and retrograde signalling. Although it is difficult to reconcile these results with our in vitro affinity measurements, one cannot discount the possibility that, under physiological conditions, the salt bridge involving the β_2a_ RT loop may be weakened, enabling the SH3 domain binding site to engage with the II-III loop.

The DHPR II-III loop residues (Ala739, Phe741, Pro742, and Asp 744) have been previously identified as being important in orthograde and retrograde coupling [12]. Our binding results show that a segment peptide comprising these II-III loop residues (SP1) interacts with the β_1a_ SH3 domain and that binding can be affected individually or collectively by altering these residues. While this series of observations does not definitively prove that the II-III loop and the β_1a_ SH3 domain participates in in vivo interactions, they do show that subtle variations in the composition of the SH3 domain plays a role in skeletal muscle EC coupling. A study employing a functionalised nanobody targeting β_1_ (nb.E8) was used to explore its effect on voltage-gated calcium channels [53]. The binding site for this nanobody was located at the SH3 domain of β_1_, with the structural complex revealing direct interactions with the RT loop and α2 helix. Addition of the nb.E8 antibody to whole cells was found to inhibit the DHPR current amplitude and modulate gating properties, supporting the proposition that the disruption of endogenous modulators (i.e., II-III loop) at this region may impact EC coupling.

### 4.2. Structural Relationship Between β_1a_ and the DHPR II-III Loop

**Figure 7:**
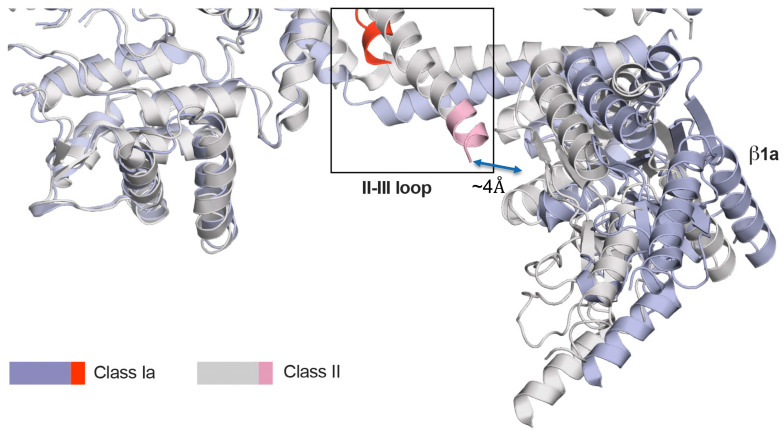
Cryo-EM structures of Cav1.1 reveals two conformational states. Overlay of two conformational states class Ia and class II, 5GJV, and 5GJW, respectively. Superposition of the DHPR II-III loop (red and light red) shows the relocation and extension of helix IIS_6_ (boxed). The II-III loop in conformation II is in close proximity (<4Å) to the α9 helix of the β_1a_ GK domain.

Due to the disordered nature of the II-III loop, no definitive structural evidence demonstrating the direct interaction of the β_1a_ SH3 domain with the II-III loop is available. However, it has been noted through a FRET study that, within the triad environment, the presence of β_1a_ modulates the α_1s_ II-III conformation [54]. Other indirect support of this association can be found in the cryo-em structure of CaV1.1 (skeletal DHPR), where the N-terminal II-III loop helix was noted to be adjacent (<4Å) to the α9 helix of the β_1a_ GK domain [55] (Figure 7), thus placing the β_1a_ SH3 domain and C region of the II-III loop in close enough proximity to interact. Lastly, a recently published article on the role of STAC3 SH3 domains revealed that, even though its interaction with the C region of the II-III loop is very important in supporting skeletal muscle EC coupling, the deletion of these domains proved to not be essential [56]. This contrasts with the deletion or substitution of the C region of the DHPR II-III loop, which has been determined to be absolutely critical for skeletal muscle EC coupling [8,10,12]. The fact that these reciprocal experiments designed to both abolish the II-III loop/STAC3 interaction resulted in different skeletal muscle EC coupling effects lends weight to the argument there may be another binding partner of the DHPR II-III loop C region that at least supports EC coupling.

## 5. Conclusions

Our results indicate that the SH3 domain of β_1a_ recognizes polyproline elements located in the cytoplasmic α_1s_ DHPR II-III loop, one of which has been demonstrated to be important for skeletal muscle EC coupling. In vitro affinity experiments using a combination of skeletal and cardiac beta and alpha subunit isoforms reveal that the interaction is influenced by the amino acid composition of the various isoforms. The findings presented here offer a unique insight into the potential signalling pathway that may contribute to skeletal muscle EC coupling. Whereas the binding partner of the α_1s_ II-III loop was previously ascribed to STAC3, we show that the SH3 domain β_1a_ subunit may form part of the communication network between the DHPR voltage-sensing subunit, leading to RyR1 activation.

## Figures and Tables

**Table 1:** Binding affinities of selected skeletal and cardiac DHPR II-III loop peptides to β_1a_ and the β_1a_ SH3 domain as measured by tryptophan fluorescence quenching.

DHPR II-III Fragment	β_1a_ Subunit (μM)	β_1a_–SH3 Domain (Residues 101–272) (μM)
α_1s_ DHPR II-III Loop	2.5 ± 0.4	2.6 ± 0.2
α_1s_ II–III loop C region (α_1s_ C)	4.9 ± 0.6	3.6 ± 0.6
α_1c_ II–III loop C region (α_1c_ C)	3.5 ± 0.4	2.5 ± 0.2
Scrambled α_1s_ C	No binding	No binding
Mutated α_1s_ C All P⟶A	No binding	No binding
α_1s_ C binding peptide 1 (sP1)	3.6 ± 0.2	2.6 ± 0.4
sP1 A⟶P mutant	4.5 ± 0.3	2.3 ± 0.2
sP1 F⟶T mutant	4.6 ± 0.4	4.6 ± 0.3
sP1 P⟶T mutant	22 ± 2	19 ± 2
sP1 D⟶E mutant	15 ± 2	17 ± 1
α_1c_ C binding peptide 1 (cP1)	14 ± 2	19 ± 4
α_1s_ C binding peptide 2 (sP2)	5.4 ± 0.9	3.8 ± 0.2
α_1c_ C binding peptide 2 (cP2)	4.6 ± 0.3	3.6 ± 0.3
α_1s_ I-II Loop	0.0152 ± 0.0018	No binding

**Table 2:** Calcium release and L-type channel parameters of myotubes expressing β_1a_ wild-type and RT-loop modifications.

	Gmax (pS/pF)	V_1/2_, mV	V_r_, mV	k, mV
Wt-β_1a_ (n = 22)	453 ± 33.0	14.7 ± 0.88	84.8 ± 0.35	6.0 ± 0.17
β_1a_-RT loop (n = 21)	521 ± 36.6	11.2 ± 0.77 **	85.6 ± 0.48	5.8 ± 0.23

** *p* = 0.0051 vs. Wt-β_1a_ (*t*-test).

**Table 3:** Binding affinities of various β isoforms and chimeras to α_1s_ C.

β-Construct	β_1a_	β_2a_	β_1a_/β_2a_ RT Loop	Β2_a_: HE to PG Mutant	β_1a_/β_2a_ RT Loop: HE to PG Mutant
**K_d_ (μM)**	4.9 ± 0.6	No binding	Weak binding	10.4 ± 0.7	8.7 ± 0.6

## Data Availability

The original contributions presented in this study are included in the article/Supplementary Materials. Further inquiries can be directed to the corresponding author.

## Supplementary Materials

The following supporting information can be downloaded at: https://www.mdpi.com/article/10.3390/biom15111610/s1, Figure S1: Purified recombinant proteins used for binding experiments; Figure S2: Circular dichroism (CD) spectra of β_1a_ subunits—(A) CD spectrum of the purified and refolded full length β_1a_ protein. The spectrum shows a maximum at 191 nm followed by minima at 209 and 226 nm. (B) CD spectrum of the purified and refolded full length SH3 domain b1a protein (101–272).
