# Supplementary material for: Interactive Role of the DHPR β1a SH3 Domain in Skeletal Muscle Excitation–Contraction Coupling
Source: Biomolecules. 2025 Nov 17;15(11):1610. doi: 10.3390/biom15111610 (PMC12650144; doi:10.3390/biom15111610)
Supplement: Supplementary file 1 [file biomolecules-15-01610-s001.zip › biomolecules-3924742-supplementary.pdf]

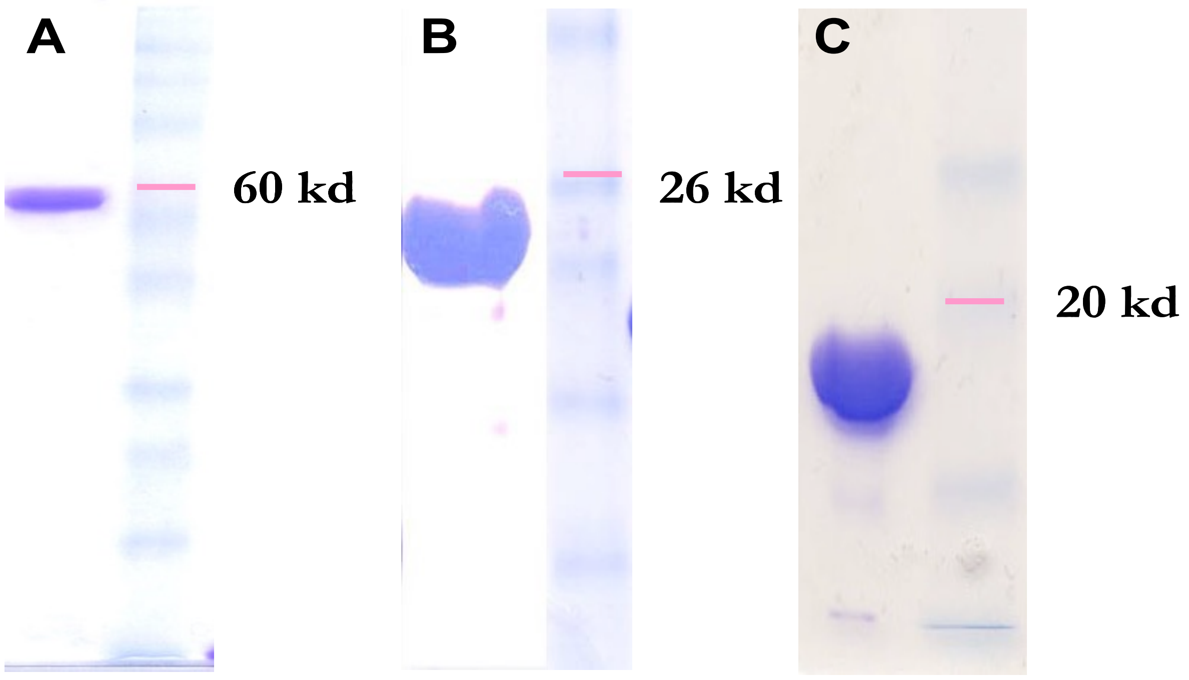

Supp Figure S1 Purified recombinant proteins used for binding experiments. 12% SDS-PAGE coomassie stained gel. **A)** – Full length β_{1a} subunit, **B)** - β_{1a}-SH3 domain, **C)** - α_{1s} II-III loop. Right hand lane of each gel is a protein marker (BenchMark™ prestained- Invitrogen). Pink band corresponds to the molecular weight noted.

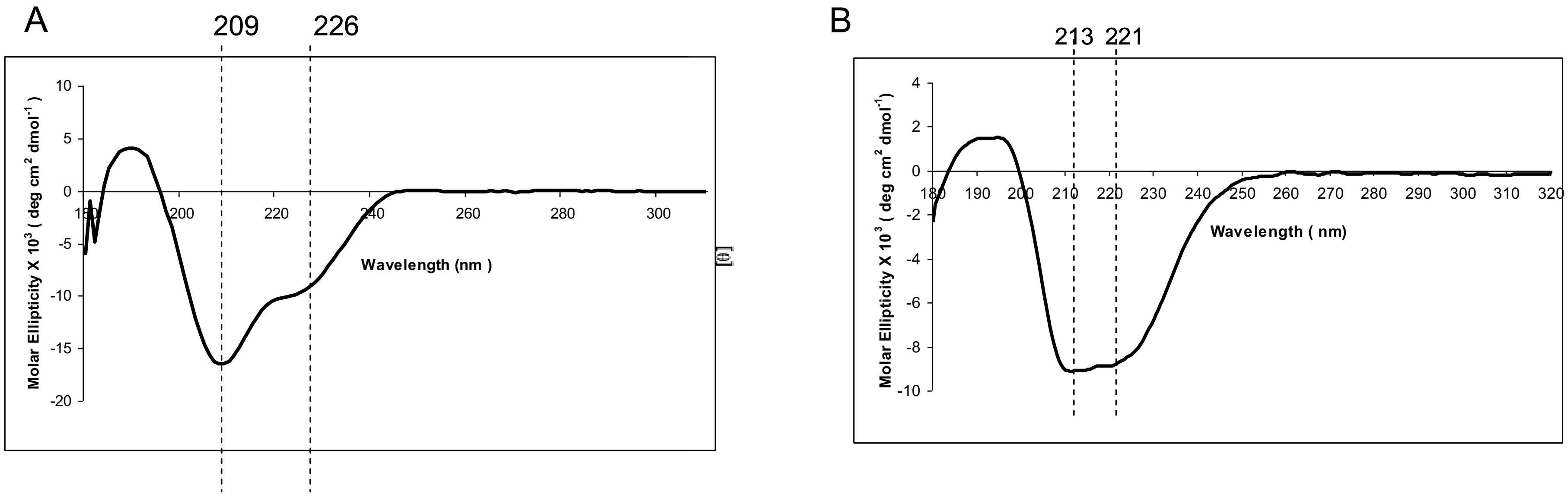

Supp Figure S2 Circular dichroism (CD) spectra of β_{1a} subunits – (A) CD spectrum of the purified and refolded full length β_{1a} protein. The spectrum shows a maximum at 191 nm followed by minima at 209 and 226 nm. (B) CD spectrum of the purified and refolded full length SH3 domain β_{1a} protein (101-272) . The spectrum shows a maximum at 192 nm followed by minima at 213 and 221 nm.
